# Detection of immunogenic cell death and its relevance for cancer therapy

**DOI:** 10.1038/s41419-020-03221-2

**Published:** 2020-11-26

**Authors:** Jitka Fucikova, Oliver Kepp, Lenka Kasikova, Giulia Petroni, Takahiro Yamazaki, Peng Liu, Liwei Zhao, Radek Spisek, Guido Kroemer, Lorenzo Galluzzi

**Affiliations:** 1 Sotio, Prague, Czech Republic; 2 2nd Faculty of Medicine and University Hospital Motol, Department of Immunology, Charles University, Prague, Czech Republic; 3 Equipe labellisée par la Ligue contre le cancer, Centre de Recherche des Cordeliers, INSERM U1138, Université de Paris, Sorbonne Université, Paris, France; 4 Metabolomics and Cell Biology Platforms, Institut Gustave Roussy, Villejuif, France; 5 Department of Radiation Oncology, Weill Cornell Medical College, New York, NY USA; 6 Pôle de Biologie, Hôpital Européen Georges Pompidou, AP-HP, Paris, France; 7 Suzhou Institute for Systems Medicine, Chinese Academy of Sciences, Suzhou, China; 8 Department of Women’s and Children’s Health, Karolinska Institute, Karolinska University Hospital, Stockholm, Sweden; 9 Sandra and Edward Meyer Cancer Center, New York, NY USA; 10 Caryl and Israel Englander Institute for Precision Medicine, New York, NY USA; 11 Department of Dermatology, Yale University School of Medicine, New Haven, CT USA; 12 Université de Paris, Paris, France

**Keywords:** Cancer immunotherapy, Drug discovery, Immune cell death

## Abstract

Chemotherapy, radiation therapy, as well as targeted anticancer agents can induce clinically relevant tumor-targeting immune responses, which critically rely on the antigenicity of malignant cells and their capacity to generate adjuvant signals. In particular, immunogenic cell death (ICD) is accompanied by the exposure and release of numerous damage-associated molecular patterns (DAMPs), which altogether confer a robust adjuvanticity to dying cancer cells, as they favor the recruitment and activation of antigen-presenting cells. ICD-associated DAMPs include surface-exposed calreticulin (CALR) as well as secreted ATP, annexin A1 (ANXA1), type I interferon, and high-mobility group box 1 (HMGB1). Additional hallmarks of ICD encompass the phosphorylation of eukaryotic translation initiation factor 2 subunit-α (EIF2S1, better known as eIF2α), the activation of autophagy, and a global arrest in transcription and translation. Here, we outline methodological approaches for measuring ICD markers in vitro and ex vivo for the discovery of next-generation antineoplastic agents, the development of personalized anticancer regimens, and the identification of optimal therapeutic combinations for the clinical management of cancer.

## Facts

- Immunogenic cell death (ICD) can initiate adaptive immune responses, because it is accompanied by the emission of adjuvant-like signals commonly known as damage-associated molecular patterns (DAMPs).
- Key DAMPs for cell death to be perceived as immunogenic include calreticulin, HMGB1, ATP, ANXA1, and type I IFN.
- DAMP emission by cells undergoing ICD often relies on the activation of intracellular responses of adaptation to stress.
- Although detecting DAMP emission from dying cells informs on the potential immunogenicity of cell death, in vivo assays are required to validate bona fide instances of ICD.

## Open questions

- Does a core set of DAMPs common to all instances of ICD exist?
- Can we harness transcriptional signatures of pattern recognition receptor (PRR) signaling to assess DAMP emission in patient biopsies?
- Will therapeutic strategies specifically conceived to restore the immunogenicity of cell death enter the clinical practice for cancer therapy?

## Introduction

The emergence and progression of human neoplasms strongly depends on the interaction between cancer cells and their microenvironment, especially in its immunological components^1–7^. Immunosurveillance is generally mediated by type 1 CD4^+^ T-helper (T_H_1) cells and CD8^+^ cytotoxic T lymphocytes (CTLs), which specifically recognize antigenic epitopes emerging during malignant transformation and tumor progression^8–13^. Specifically, neoplastic cells can become visible to the adaptive immune system as a consequence of non-synonymous mutations in the coding region of actively expressed genes (leading to antigens that are not covered by central tolerance) or the ectopic expression of otherwise normal antigens for which central tolerance is leaky^9,14–17^. Thus, the (neo)antigenic profile of a tumor is a key determinant of anticancer immune responses^18–20^, as demonstrated by the fact that several solid tumors become resistant to immunotherapy with immune checkpoint inhibitors (ICIs) by acquiring defects in the antigen presentation machinery^21–25^. However, antigenicity is not sufficient for malignant cells to initiate anticancer immunity, as antigen presentation by dendritic cells (DCs) in the absence of co-stimulatory signals to T cells generally results in the establishment of immunological tolerance^26–30^. Thus, malignant cells can also escape immunosurveillance by losing their capacity to promote the recruitment and functional maturation of DCs or their precursors, a feature that is cumulatively referred to as adjuvanticity^31–33^. Although the adjuvanticity of infected cells mostly originates from microbial products commonly referred to as microbe-associated molecular patterns (MAMPs; e.g., lipopolysaccharide)^34–36^, malignant cells mediate chemotactic and immunostimulatory effects by emitting the so-called damage-associated molecular patterns (DAMPs) and secreting cytokines as they adapt or succumb to microenvironmental perturbations^16,37,38^.

Immunogenic cell death (ICD) represents a functionally unique response pattern that comprises the induction of organellar and cellular stress, and culminates with cell death accompanied by the exposure, active secretion, or passive release of numerous DAMPs^16,37,39–41^. The spatiotemporally defined emission of DAMPs in the course of ICD and their binding to specific pattern recognition receptors (PRRs) expressed by DCs initiates a cellular cascade that ultimately results in the activation of both innate and adaptive immune responses^34,42–44^. In line with this notion, accumulating preclinical and clinical evidence indicates that various DAMPs and DAMP-associated processes may have prognostic and predictive value for patients affected by a variety of tumors^45^. Moreover, there is ample evidence that treatment-driven ICD can elicit anticancer immune responses that reinforce the therapeutic effects of conventional anticancer chemotherapies and radiotherapy^46–49^. So far, however, only a few bona fide ICD inducers have been successfully employed in the clinic as therapeutics (Table 1)^46,50^. These agents may be particularly relevant to initiate anticancer immune responses that can be actioned by ICIs or other forms of immunotherapy in the context of combinatorial treatment regimens^46,51–55^, as demonstrated in some clinical^56,57^ and numerous preclinical^58–60^ studies. In line with this notion, numerous Food and Drug Administration-approved ICD inducers are currently being investigated in off-label oncological settings, especially in combination with ICIs or other immunotherapeutics^61–65^.

**Table 1:** Immunogenic cell death inducers commonly employed as conventional chemotherapeutics^a^.

Agent	eIF2α phosphorylation	DAMPs	Main ICD-associated cytokines	Combination with ICIs in mice
Anthracyclines	Yes	ANXA1 ATP CALR HMGB1	Type I IFN CXCL10 IL-1β IL-17	Anti-PD-1 Anti-CTLA4
Bleomycin	Yes	ATP CALR HMGB1	NA	NA
Bortezomib	Yes	CALR HSP70	NA	NA
Cyclophosphamide	NA	ATP CALR HMGB1	Type I IFN IL-17	Anti-PD-1 Anti-CTLA4
Dactinomycin	Yes	ATP CALR HMGB1	Type I IFN IL-17	Anti-PD-1
Lurbinectedin	Yes	ATP CALR HMGB1	Type I IFN	Anti-PD-1 Anti-CTLA4
Oxaliplatin	Yes	ATP CALR HMGB1 HSP70	Type I IFN IL-1β	Anti-PD-1 Anti-CTLA4
Teniposide	NA	CALR HMGB1	Type I IFN	Anti-PD-1

*ANXA1* annexin A1, *CALR* calreticulin, *CTLA4* cytotoxic T lymphocyte-associated protein 4, *eIF2α* (official name: EIF2S1) eukaryotic translation initiation factor 2 subunit-α, *HMGB1* high-mobility group box 1, *HSP70* (official name: HSPA1A), heat-shock protein family A (Hsp70) member 1A, *IFN* interferon, *IL-1β* (official name: IL1B), interleukin 1 beta, *IL-17* (official name: IL17), interleukin 17, *NA* not available, *PD-1* (official name: PDCD1) programmed cell death 1.

^a^Adapted from ref. ^46^, not including targeted anticancer agents and extracorporeal photochemotherapy.

Thus, the development of methodological approaches and platforms for identifying novel ICD inducers should accelerate the development of next-generation anticancer therapeutics, ultimately improving the clinical management of a large population of oncological patients.

## Main hallmarks of ICD

ICD can be induced by different stressors, including but not limited to (1) intracellular pathogens^66–68^; (2) conventional chemotherapeutics such as anthracyclines, DNA-damaging agents, and proteasomal inhibitors^50,69–72^; (3) targeted anticancer agents such as the tyrosine kinase inhibitor crizotinib, the epidermal growth factor receptor-specific monoclonal antibody cetuximab and poly-ADP-ribose polymerase (PARP) inhibitors^59,73–76^; and (4) numerous physical modalities, encompassing hypericin- and redaporfin-based photodynamic therapy, extracorporeal photochemotherapy, various forms of ionizing radiation, high hydrostatic pressure, and severe heat shock^77–81^.

**Fig. 1:**
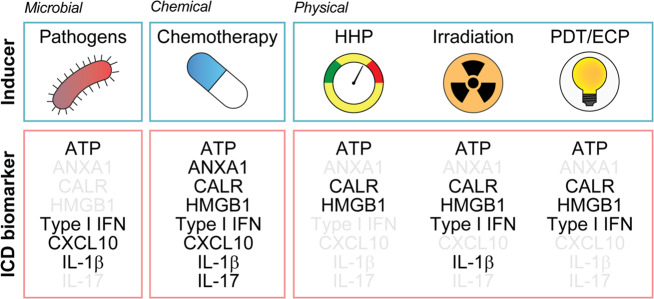
Main hallmarks of ICD. Different inducers of immunogenic cell death (ICD) have been shown to elicit incompletely overlapping molecular signatures with respect to ICD biomarkers. This not only reinforces the need for the simultaneous assessment of multiple surrogate ICD biomarkers in the context of screening campaigns, but also identifies an originally unsuspected diversity in the molecular and cellular mechanisms supporting adaptive immunity downstream of danger signaling. ANXA1, annexin A1; CALR, calreticulin, CXCL10, C-X-C motif chemokine ligand 10; ECP, extracorporeal photochemotherapy; HHP, high hydrostatic pressure; HMGB1, high-mobility group box 1; IFN, interferon; IL-1β (official name: IL1B), interleukin 1 beta; IL-17 (official name: IL17), interleukin 17; PDT, photodynamic therapy.

DAMPs emitted in the course of ICD include endoplasmic reticulum (ER) chaperones such as calreticulin (CALR) and heat-shock proteins (HSPs), which are exposed on the cell surface, the non-histone chromatin-binding protein high-mobility group box 1 (HMGB1), the cytoplasmic protein annexin A1 (ANXA1), and the small metabolite ATP that are liberated from dying cells into the extracellular space, as well as type I interferons (IFNs) that are released upon de novo synthesis^38,82–84^. DAMPs can be recognized by both the innate and adaptive immune systems via distinct PRRs driving chemoattraction, homing, activation, and/or maturation, ultimately resulting in the cross-presentation of tumor antigens to CD8^+^ CTLs in the context of robust immunostimulation^34,43^. Other hallmarks of ICD include the phosphorylation of eukaryotic translation initiation factor 2 subunit-α (EIF2S1, better known as eIF2α), the activation of autophagy, and a global arrest in transcription and translation^85–88^. Importantly, not all ICD inducers activate the same stress responses and hence elicit the same molecular signals^16^. Thus, for instance, although autophagy is strictly required for anthracycline-driven cancer cell death to be perceived as immunogenic^86^, the same does not hold true for the demise of cancer cells exposed to ionizing radiation^89^ (Fig. 1).

### Calreticulin

CALR exposed on the plasma membrane of malignant cells undergoing ICD serves as an “eat-me” signal that facilitates the engulfment of dying cells or their corpses by DCs or their precursors, thus providing them with an abundant source of antigenic material^90–93^. The molecular mechanism underlying the ICD-associated exposure of CALR include (1) the phosphorylation of eIF2α, accompanied by (2) a robust arrest of protein translation and (3) the activation of pro-apoptotic caspase 8 (CASP8), followed by the cleavage of B-cell receptor-associated protein 31 (BCAP31), the aggregation of the pro-apoptotic Bcl-2 family members BCL2-associated X protein (BAX) and BCL2-antagonist/killer 1 (BAK1) at the outer mitochondrial membrane, and (4) the vesicle-associated membrane protein 1 (VAMP1)- and synaptosome-associated protein 25 (SNAP25)-mediated anterograde transport to the Golgi apparatus and exocytosis^94^.

Surface-exposed CALR binds to LDL-receptor-related protein 1 (LRP1, best known as CD91), which is the main ER chaperone-sensing PRR expressed by antigen-presenting cells including DCs^95,96^. CD91 ligation promotes the engulfment of cellular corpses and debris by a mechanism that depends on the GTPase Rac family small GTPase 1 (RAC1)^78,95,97^. Consistent with the key role of CALR exposure in the perception of cell death as immunogenic, CALR knockdown by RNA interference (RNAi), *CALR* deletion by CRISPR/Cas9, or CALR blockade by neutralizing antibodies decreases the potency of ICD-mediated anticancer immune responses in a variety of settings^79^. Besides the role of CALR on the initiation of adaptive T-cell-mediated immunity downstream of ICD, we have recently demonstrated that CALR spontaneously exposed on the surface of malignant blasts from acute myeloid leukemia patients is associated with improved innate immunity as a consequence of improved interleukin 15 (IL15) *trans*-presentation to natural killer (NK) cells^98,99^. Altogether, these findings document that surface-exposed ER chaperones are important hallmarks of ICD stimulating both innate and adaptive anticancer immunity.

### High-mobility group box 1

The release of HMGB1 from cancer cells undergoing ICD involves the permeabilization of both the nuclear lamina and the plasma membrane in a two-step process that enables the translocation of the protein from the nucleus to the cytoplasm, followed by its liberation into the extracellular space^100,101^. Extracellular HMGB1 can bind multiple PRRs expressed by myeloid cells, encompassing advanced glycosylation end-product-specific receptor (AGER, best known as RAGE) and Toll-like receptor 4 (TLR4)^102–105^. However, it appears that TLR4 signaling via MYD88 innate immune signal transduction adaptor (MYD88)^106–108^ is required and sufficient for cell death to be perceived as immunogenic, as demonstrated with a variety of genetic and pharmacological approaches^16^. In line with this notion, the knockout of *HMGB1* in cancer cells and the antibody-mediated neutralization of TLR4 in the host limit therapeutically relevant immune responses (and hence disease control) driven by anthracyclines, cyclophosphamide, or oxaliplatin in preclinical in vivo models^103,109^. In addition, loss-of-function polymorphisms in *TLR4* have been associated with unfavorable disease outcome in patients with breast cancer receiving anthracyclines as part of their clinical management^103^, in head and neck squamous cell carcinoma patients exposed to standard-of-care chemotherapy^110^, as well as in melanoma patients treated with an experimental DC-based vaccine^111,112^. Altogether, these findings indicate that the HMGB1-mediated activation of TLR4 is a crucial constituent of ICD-elicited immunogenicity. That said, HMGB1 release appears to be common to both immunogenic and non-immunogenic variants of cell deaths. In line with this notion, extracellular HMGB1 has been consistently used as a biomarker for plasma membrane permeabilization^113^. This implies that the mere detection of HMGB1 release cannot be interpreted as a reliable sign of ICD.

### ATP

During the course of ICD, ATP is released in an autophagy-dependent manner through the active exocytosis of ATP-containing vesicles via pannexin channels^114–116^. Extracellular ATP operates as a prominent “find-me” signal for DC precursors and macrophages upon binding to purinergic receptor P2Y2 (P2RY2, a metabotropic receptor), thus facilitating the recruitment of myeloid cells to sites of active ICD^117,118^. Moreover, extracellular ATP mediates pro-inflammatory effects upon activation of the CASP1-dependent NLRP3 inflammasome and consequent secretion of mature interleukin 1 beta (IL1B, best known as IL-1β) and IL-18^119–121^. These effects, which originate from purinergic receptor P2X 7 (P2RX7, an inotropic receptor), culminate with the activation of CD8^+^ T cells and IL-17-producing γδ T cells^119^. Consistent with the importance of these events for immune responses driven by ICD, the immunogenicity of cell death is abrogated when either ATP fails to accumulate in the microenvironment of dying cancer cells or when P2RX7 or P2RY2 are absent from the myeloid compartment of the host^119^. Thus, overexpression of the ATP-degrading ectoenzyme ectonucleoside triphosphate diphosphohydrolase 1 (ENTPD1, best known as CD39) in malignant tissues exerts potent immunosuppressive effects^122–124^. Moreover, loss-of-function polymorphisms in *P2RX7* has been associated with poor clinical outcome in breast cancer patients subjected to anthracycline-based chemotherapy^119^. Of note, the cell death-associated secretion of ATP resembles HGMB1 release in that it can also accompany non-immunogenic variants of cell death. At least in vitro, however, normalizing extracellular ATP levels to percentage of dead cells or to the ATP plateau that can be achieved with a detergent aids the discrimination of non-immunogenic *vs*. immunogenic forms of cell death.

### Annexin A1

The mechanisms involved in the release of ANXA1 by cancer cells succumbing to ICD remain largely obscure. However, ANXA1 appears to mediate a non-redundant role as a homing factor that governs the final approach of DCs or their precursors to malignant cells undergoing ICD^125^. Consistent with such a key function, malignant cells lacking *Anxa1* exhibit limited sensitivity to anthracycline-based chemotherapy in vivo^125^. Similarly, the anticancer activity of anthracyclines in mice is amply compromised when the host lacks formyl peptide receptor 1 (FPR1), which is the main receptor for extracellular ANXA1^125^. Moreover, loss-of-function polymorphisms in *FPR1* have been associated with poor overall survival and metastasis-free survival in breast cancer patients receiving adjuvant anthracycline-based chemotherapy^125^.

### Type I interferon

Finally, ICD is accompanied by a robust type I IFN response, which can be driven by both RNA and DNA species^126–128^. In the former setting, the receptor is endosomal TLR3^126,129^, whereas in the latter scenario a key role is played by cytosolic cyclic GMP-AMP synthase (CGAS) and its signal transducer stimulator of IFN response cGAMP interactor 1 (STING1, best known as STING)^89,130,131^. Irrespective of the precise mechanism underlying type I IFN secretion, this cytokine mediates prominent immunostimulatory effects upon binding to homodimeric or heterodimeric receptors expressed by various immune cells^36,132,133^. For instance, type I IFN is known to enhance the cytotoxic functions of both CD8^+^ T cells and NK cells^134^, and promote cross-priming by DCs^135,136^. Moreover, type I IFN can trigger the secretion of pro-inflammatory mediators by macrophages^137^ and inhibit the immunosuppressive functions of CD4^+^CD25^+^FOXP3^+^ regulatory T cells^138^. Besides these direct immunostimulatory functions, type I IFN also elicits the synthesis of the C-X-C motif chemokine ligand 10 (CXCL10, a prominent chemotactic factor) by cancer cells undergoing ICD via an autocrine signaling loop^126^. The immunogenicity of ICD driven by anthracyclines and radiation therapy strongly relies on type I IFN signaling, as documented by the fact that therapeutic efficacy in mice is amply reduced when neoplastic lesions key components of the type I IFN response, such as *Ifnar1*, *Ifnar2*, *Tlr3*, *Cgas*, or *Sting1*, as well as when mice are co-treated with IFNAR1-blocking antibodies^126,139,140^. Along similar lines, *Ifnar1*^*−/−*^ neoplastic cells exposed to doxorubicin in vitro lost their capacity to vaccinate syngeneic hosts against a rechallenge with living cells of the same type due their inability to prime adaptive immune responses^126^. That said, although acute, robust type I IFN responses have been consistently associated with immunostimulation, chronic, indolent type I IFN signaling mediates immunosuppressive effects^83^. Thus, caution should be employed when characterizing type I IFN responses in the context of ICD.

Irrespective of this and other caveats, DAMP-dependent adjuvanticity occupies a key position in the mechanism that governs the immunogenicity of malignant cells succumbing to ICD.

## Monitoring CALR, HSPs, and the ISR

The exposure of CALR and other ER chaperones such as heat-shock protein family A (Hsp70) member 1A (HSPA1A, best known as HSP70) and heat-shock protein 90 α-family class A member 1 (HSP90AA1, best known as HSP90) on the plasma membrane of cells undergoing ICD can be monitored by several assays. The cytofluorometric detection of CALR exposure requires the use of specific anti-CALR antibodies and vital dyes such as such as 4′,6-diamidino-2-phenylindole (DAPI), propidium iodide (PI), or 7-aminoactinomycin D (7-AAD), to exclude permeabilized cells from the analysis and hence to avoid false-positive values^98,99^. The transgene-enforced expression of a CALR-HaloTag™ fusion protein^141,142^ can be also be used to specifically detect CALR exposure on (hitherto) living cells based on a cell-impermeant fluorescent HaloTag™ ligand^143^. However, this approach requires transgenic cell lines and hence is not suitable for ex vivo applications on freshly collected malignant cells. Surface-exposed CALR and other ER chaperones can also be detected by immunoblotting after cell surface proteins are biotinylated in pre-apoptotic cells (to avoid the detection of intracellular chaperones), followed by streptavidin-mediated precipitation^78^. Alternatively, fluorescence microscopy can be harnessed to monitor subcellular CALR localization, either upon immunostaining with CALR-specific antibodies, or in cells that have been engineered to express CALR in conjunction with a fluorescent moiety^144,145^. The latter technology is particularly advantageous for high-content screening (HCS) campaigns aimed at the identification of agents that cause CALR/HSP translocation on the plasma membrane. Retrospectively monitoring CALR/HSP expression in formalin-fixed paraffin-embedded bioptic samples from cancer patients by immunohistochemistry coupled to the evaluation of clinicopathological variables, offers a tool to estimate the impact of CALR/HSP exposure on disease progression^91,146^. Nevertheless, this technique is unable to precisely distinguish between the intracellular and surface-exposed pools of CALR and HSPs.

The ICD-associated exposure of CALR/HSP depends on the so-called integrated stress response (ISR), which is orchestrated around the inactivating phosphorylation of eIF2α^147,148^. The latter is generally catalyzed by eukaryotic translation initiation factor 2α kinase 2 (EIF2AK2, best known as PKR) and EIF2AK3 (best known as PERK), which are particularly sensitive to the accumulation of unfolded proteins within the ER^149^. Intriguingly, the other reticular arms of the ISR such as the splicing of X-box binding protein 1 (*XBP1*)^150^, as well as the derepression of activating transcription factor 4 (ATF4) and ATF6^151,152^ are not mechanistically linked to the immunogenicity of dying cancer cells, meaning that solely the phosphorylation of eIF2α constitutes a pathognomonic feature of ICD^149,153^. The ICD-associated phosphorylation of eIF2α can be detected by immunoblotting, flow cytometry, and immunofluorescence microscopy based on phosphoneoepitope-specific antibodies^154–158^, with the latter two approaches offering the scalability that is needed for HCS applications.

## Monitoring HMGB1 release

The ICD-associated release of HMGB1 can be evaluated indirectly, upon quantification of the residual pool of intracellular HMGB1 by immunoblotting, as well as directly, upon assessment of extracellular HMGB1 levels in cell culture supernatants based on commercially available enzyme-linked immunosorbent assay (ELISA) kits^80,103,143,159–162^. ELISA kits are advantageous in that they offer a precise and sensitive means to quantify HMGB1 in a variety of samples including culture supernatants, sera and other biological fluids^163–165^. An alternative technological approach consists in the generation of cells expressing a green fluorescent protein (GFP)-tagged variant of HMGB1, which can be assessed by fluorescence microscopy in the presence of an appropriate nuclear counterstain, to quantify the residual pool of nuclear HMGB1^160^. This approach offers adequate scalability for HCS applications, but obviously cannot be employed to retrospectively investigate HMGB1 release from patient samples. Immunohistochemistry has been successfully harnessed to such aim, although (at least in some setting) a clear distinction between nuclear and cytoplasmic HMGB1 has been relatively hard to make^166–168^. Recently, the retention using selective hooks (RUSH) system^169,170^ has also been established as a fully automated technology with high-throughput workflow to determine the presence of DAMPs in distinct subcellular compartments. In the RUSH system, a streptavidin-NLS3 fusion protein is used as a nuclear hook to sequestrate a streptavidin-binding peptide (SBP) fused with a target and a reporter such as HMGB1 and GFP, respectively^160^. In this setting, the exogenous addition of biotin competitively disrupts the interaction between streptavidin-NLS3 and HMGB1-SBP-GFP to release the biosensor from its hook, hence allowing the fluorescent signal to leave the nucleus provided that an HMGB1-releasing stimulus is present^160^. The main advantage of the RUSH system is that it limits the amount of false-positive hits due to auto-fluorescent molecules and enables the retention of HMGB1 unless biotin is provided, constituting an interesting investigational platform to assess ICD-related processes specifically linked to HMGB1 release in the context of near-to-normal HGMB1 levels (which is not the case for RNAi- or CRISPR/Cas9-based manipulations).

## Monitoring ATP secretion

In analogy to HMGB1, ICD-associated secretion of ATP can be monitored both directly, upon quantification of extracellular ATP, and indirectly, upon assessment of the residual pool of intracellular ATP (after cell lysis)^86^. Commercial luminescence-based assays represent the gold standard for both direct and indirect quantification of ATP levels^171,172^. Indeed, luciferase can catalyze the oxidation of its substrate luciferin, which is associated with light emission, only in the presence of magnesium, oxygen, and ATP^173,174^, which can be harnessed for quantitative assessments based on a conventional standard curve. The main disadvantage of the direct approach reflects the potential expression of ATP-degrading enzymes such as CD39 by cancer cells^175,176^, which may lower ATP concentrations below limit-of-detection. The indirect approach may potentially be confounded by agents that alter intracellular ATP levels in the absence of any cytotoxicity (and hence any ATP release), such as drugs targeting bioenergetic metabolism^177–179^. As an alternative to luminescence-based approaches, intracellular ATP-containing vesicles can be visualized and quantified by quinacrine^180^, a fluorochrome that emits in green in the presence of ATP, enabling quantitative assessment by flow cytometry and fluorescence microscopy^181,182^.

## Monitoring the release of type I IFNs

The secretion of type I IFN from cancer cells undergoing ICD can be monitored by several assays^183^. In this setting, ELISA-based detection represents the gold standard approach, as it enables the quantitative assessment of type I IFN in a wide panel of biological specimens with superior sensitivity^184–186^. However, ELISAs are disadvantageous in that they cannot be harnessed to precisely identify type I IFN-producing cells within heterogeneous cell populations^187,188^. Such a disadvantage can be overcome by cytofluorometric tests based on intracellular staining with a type I IFN-specific (most often IFNB1-specific) antibody^98^. This approach can be widely used to analyze the production of type I IFN in cultured cells, as well as in primary tumor cells freshly isolated from patients^91^, although it is intrinsically unapt for the assessment of actual type I IFN secretion. RT-PCR and immunoblotting on cell lysates are also commonly employed to monitor type I IFN expression in cells responding to stress^139,189^. However, neither of these approaches can be employed to evaluate type I IFN secretion (as opposed to intracellular expression). Moreover, mRNA measurements do not formally evaluate type I IFN signaling, as transcription is not necessarily associated with translation^190,191^. Immunostaining based on type I IFN-specific antibodies coupled with immunohistochemistry or immunofluorescence microscopy has also been successfully employed to detect type I IFN in bioptic specimens from cancer patients and mice^192–198^. However, it is complex to discriminate between intracellular expression and secretion on these technical platforms. As an alternative to direct type I IFN measurements, genes expressed by cells exposed to type I IFN (which are commonly referred to as IFN-stimulated genes, ISGs), including MX dynamin-like GTPase 1 (MX1), have been evaluated by RT-PCR as proxies for the transcriptional response driven by IFN receptor dimers^126,199^. This approach overcomes several of the aforementioned limitations, although it cannot be implemented on a per cell basis. To this aim, although, biosensor cell lines expressing GFP under the control of the MX1 promoter have been engineered^200^. Such cells are amenable not only to cytofluorometric studies, but also to plate-based fluorescence measurements for HCS applications. A similar strategies relying on type I IFN signaling effectors has been adopted for the immunohistochemical evaluation of type I IFN activity in patient biopsies^201,202^.

## Assessment of transcription and translation

One salient feature of ICD is the inhibition of RNA transcription^85^, constituting yet another feature that can be monitored in screening campaigns aimed at the identification of novel ICD inducers. Stalled RNA synthesis can be assessed in vitro by means of a chemically derivatized uridine analog that incorporates into nascent RNA and can be visualized as a fluorescent signal by click chemistry^85^. Alternatively, the inhibition of transcription can be accessed via the immunofluorescence microscopy-based detection of nucleolin and fibrillarin, two proteins that colocalize in the nucleus when RNA synthesis is active, yet can be detected as separate entities when transcription is stalled^85^. Besides laborious methods based on the incorporation of radiolabeled amino acids into nascent proteins^203^, translational proficiency can be assessed by polysome profiling, which is commonly based on the separation of cellular lysates on a sucrose gradient coupled to immunoblotting for ribosomal subunits on the fractions collected therefrom^204,205^. It may be difficult, however, to scale up polysome profiling for HCS applications. Irrespective of these and other unresolved issues, incorporating the assessment of transcriptional and translational proficiency into screening campaigns aimed at identifying novel ICD inducers may limit false positivity rate.

## Discovery platform for the identification of ICD inducers

**Fig. 2:**
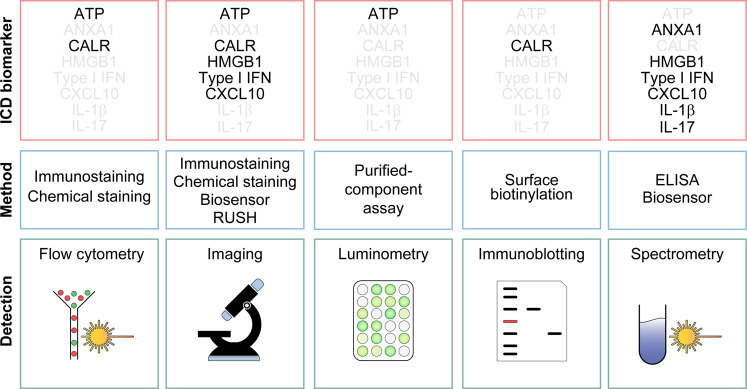
Main methodological approaches to measure ICD biomarkers in vitro. The main hallmarks of immunogenic cell death (ICD) can be assessed by flow cytometry, (immuno)fluorescence microscopy, immunoblotting, or luminometry, based on a variety of different approaches. ANXA1, annexin A1; CALR, calreticulin, CXCL10, C-X-C motif chemokine ligand 10; HMGB1, high-mobility group box 1; IFN, interferon; IL-1β (official name: IL1B), interleukin 1 beta; IL-17 (official name: IL17), interleukin 17; RUSH, retention using selective hooks.

To address the need for novel ICD-inducing agents, we have built a phenotypic screening platform that incorporates many of the aforementioned assays coupled to automated epifluorescence microscopy (Fig. 2). Specifically, we employ biosensor cell lines to measure fluorescent surrogate markers for ATP release (with quinacrine), CALR exposure (using cells stably expressing CALR-GFP), type I IFN signaling (with cells expressing GFP under the control of the *MX1* promoter), and HMGB1 release (in cells stably transduced with an HMGB1-GFP fusion) along with morphological traits of cell death such as the rarefaction of cells or the appearance of pyknotic nuclei^206^. These biosensors can be cultured in the presence of agents from large chemical collections and screened for ICD manifestations in a semi-automated manner, followed by in vitro validation experiments with alternative methods for ICD detection and additional cell lines. Finally, potential ICD inducers selected from the phenotypic screening need to be validated for their capacity to induce anticancer immune responses in vivo, in mouse models of prophylactic vaccination or therapeutic challenge. In some cases, indeed, abundant DAMP emission does not necessarily correlate with the ability of dying cells to drive anticancer immunity^143^ and in vivo functional assays remain the gold standard approach to identify bona fide ICD. More recently, we have used artificial intelligence to design algorithms that relate physicochemical descriptors of chemical agents with biological activity. These algorithms can predict the likelihood of distinct molecules to induce ICD, hence enabling the pre-selection of drugs with a high probability to operate as bona fide ICD inducers and hence reducing the cost of screening campaigns^149,206^.

## Concluding remarks

**Fig. 3:**
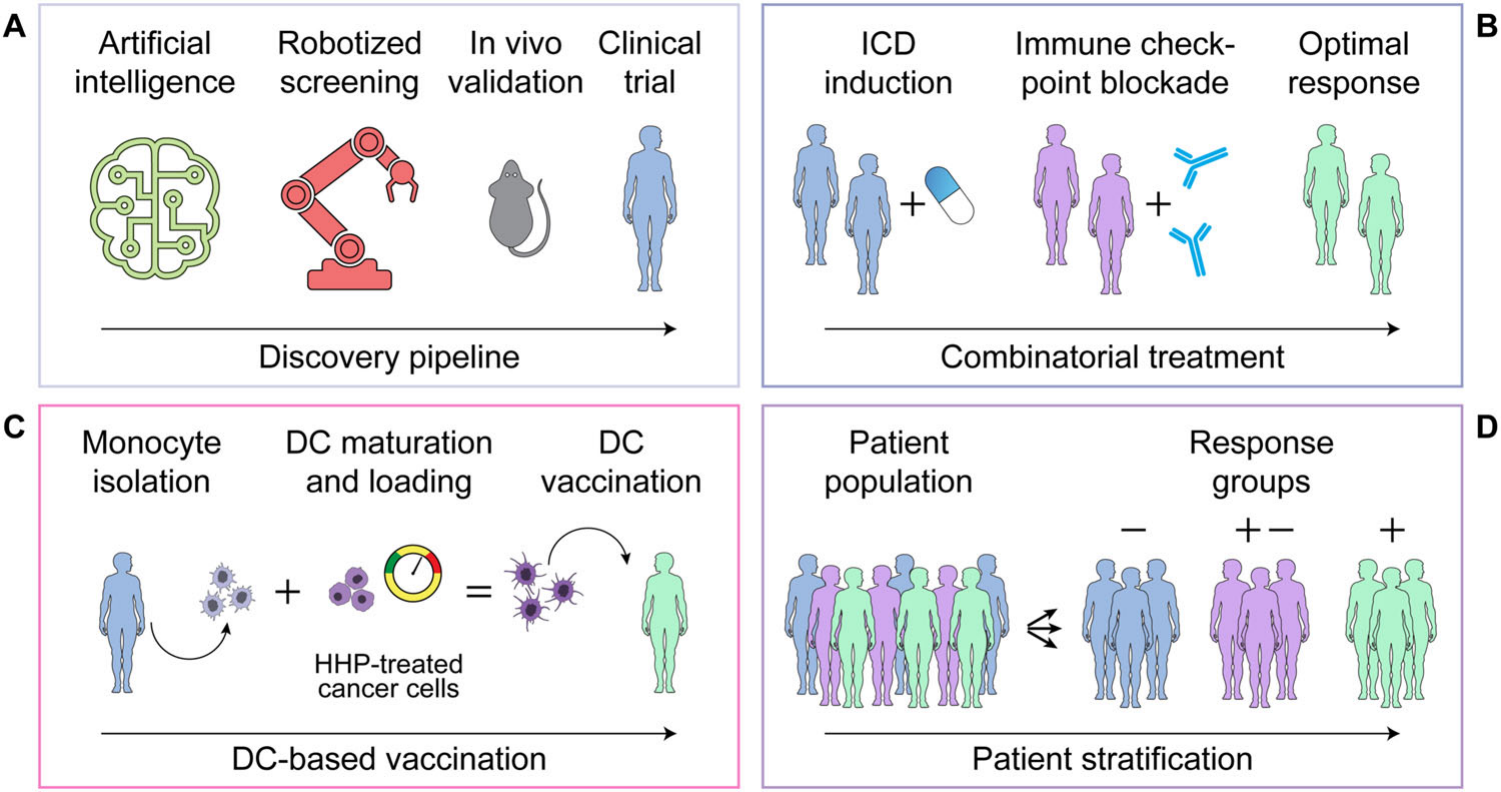
ICD inducers from HCS campaigns to the bedside. Several high-content screening (HCS) campaigns have led to the discovery of novel immunogenic cell death (ICD) inducers that have entered clinical testing, either alone (**A**), or combined with immune checkpoint inhibitors (**B**). ICD induction is also being harnessed for the generation of dendritic cell (DC)-based vaccines for therapeutic purposes (**C**). Finally, biomarkers of ICD may be used to stratify patient populations and hence identify individuals with an elevated likelihood to respond to treatment and/or subjects that would benefit from strategies correcting existing defects in ICD signaling (**D**). HHP, high hydrostatic pressure.

In summary, we and others have developed assays to assess ICD parameters in vitro (in cell cultures) and ex vivo (on tumor biopsies), which can be harnessed for the discovery of next-generation anticancer agents and the identification of optimal therapeutic regimens for clinical application, respectively. The construction of a multistep discovery pipeline involving artificial intelligence-driven pre-selection and a robotized workflow for the detection of surrogate ICD biomarkers enables us to implement various HCS campaigns that ultimately identified novel ICD inducers^206^. Some of the compounds identified with this platform have entered clinical trials, either as single agents or in combination with ICIs^61,63^. Similarly, novel approaches to drive ICD have been harnessed for the development of therapeutic DC-based vaccines, which are currently under clinical evaluation^207,208^. Moreover, the systematic assessment of ICD biomarkers such as the expression of CALR or HMGB1 on tumor biopsies may yield useful information for patient stratification (Fig. 3). That said, DAMP detection in patient samples remains particularly challenging^16^, as even in the case of proteins (which are considerably more stable than ATP), expression levels do not necessarily relate to emission^16^. At least in part, this problem could be circumvented by concomitantly assessing: (1) the intracellular levels of a specific DAMP (when possible, as for proteins) and/or the activation of the intracellular stress response that drives the emission of such DAMP (together, assessing the probability of DAMP emission); and (2) transcriptional programs driven by PRR activated by the same DAMP (as a measure of active signaling). To the best of our knowledge, however, such a combinatorial approach has not yet been undertaken.

On theoretical grounds, the absence or limited availability of ICD biomarkers should prompt the use of therapeutic approaches that (attempt to) compensate for the missing factors^209^. For instance, the absence of CALR might be compensated by the direct injection of recombinant CALR into the tumor or the administration of a CD47-blocking antibody, which neutralizes the main functional antagonist of CALR^210,211^. Similarly, the absence of HMGB1 might be compensated by the administration of recombinant HMGB1 itself or alternative TLR4 agonists^212^. These examples illustrate how the in-depth exploration of ICD-related processes and molecules might yield knowledge that may be harnessed to improve cancer therapies in a personalized, biomarker-driven manner.
